# ICU-Managed Patients' Epidemiology, Characteristics, and Outcomes: A Retrospective Single-Center Study

**DOI:** 10.1155/2023/9388449

**Published:** 2023-01-17

**Authors:** Ahmed Muhammad Bashir, Marian Muse Osman, Hawa Nuradin Mohamed, Ifrah Adan Hilowle, Halima Abdulkadir Ahmed, Abdirahman Abdikadir Osman, Osman Abubakar Fiidow

**Affiliations:** ^1^Department of Internal Medicine, Mogadishu Somali Turkey Training and Research Hospital, Mogadishu, Somalia; ^2^Department of Research, National Institute of Health Somalia, Mogadishu, Somalia; ^3^Department of Education, Mogadishu Somali Turkey Training and Research Hospital, Mogadishu, Somalia; ^4^Department of Anesthesia, Mogadishu Somali Turkey Training and Research Hospital, Mogadishu, Somalia; ^5^School of Public Health and Research, Somali National University, Mogadishu, Somalia; ^6^Somali Center for Research and Consultancy, Mogadishu, Somalia

## Abstract

**Background:**

Resources are limited, and it is exceedingly difficult to provide intensive care in developing nations. In Somalia, intensive care unit (ICU) care was introduced only a few years ago.

**Purpose:**

In this study, we aimed to determine the epidemiology, characteristics, and outcome of ICU-managed patients in a tertiary hospital in Mogadishu.

**Methods:**

We retrospectively evaluated the files of 1082 patients admitted to our ICU during the year 2021.

**Results:**

The majority (39.7%) of the patients were adults (aged between 20 and 39 years), and 67.8% were male patients. The median ICU length of stay was three days (IQR = 5 days), and nonsurvivors had shorter stays, one day. The mortality rate was 45.1%. The demand for critical care services in low-income countries is high.

**Conclusion:**

The country has a very low ICU bed capacity. Critical care remains a neglected area of health service delivery in this setting, with large numbers of patients with potentially treatable conditions not having access to such services.

## 1. Introduction

Despite continuing to be one of the world's lowest-income countries, Somalia has improved its healthcare over the last two decades. Its citizens' life expectancy has increased from 51 years in 2000 to 58 years in 2020 [1]. However, until recently, there had been little investment in secondary and tertiary care services.

Given the disproportionate burden of diseases like malaria, tuberculosis, HIV/AIDS, and trauma, the prevalence of critical illness in developing countries is disproportionately high. 25% of the world's disease burden is carried by sub-Saharan Africa [2]. Critically ill patient management necessitates substantial human, material, and financial resources. Low-income nations like Somalia often have fewer of these resources. Large hospitals in urban or metropolitan settings are the most common places to find major intensive care units (ICUs) [3].

The intensive care unit (ICU) facility is a significant and expensive part of modern healthcare. With extensive ICU infrastructures in all developed countries, the number of ICU beds available varies greatly between and among nations [4].

Evaluating the characteristics and outcomes of critically ill patients admitted to ICUs in low-income nations may contribute to determining the priorities and resources needed to enhance the care of critically ill patients in resource-constrained areas of the world. So, in this study, we aimed to determine the characteristics, admission diagnoses, and outcomes of patients admitted to the Mogadishu Somali Turkish Training and Research Hospital ICU from January 2021 until December 2021. The information collected will be used by other ICUs in the country to improve services and assist institutions that are establishing new ICUs, and the findings are believed to add something valuable to the growing amount of research showing the differences between critical care in high- and low-income nations.

## 2. Methods

This retrospective study was approved by the Institutional Review Board of Mogadishu Somali Turkey Training and Research Hospital. The health information system was reviewed, and anonymity was preserved for each case record. We evaluated all adults patients admitted to the intensive care units from January 2021 to December 2021.

Data analysed during this study came from the demographics, characteristics, and outcomes of these patients admitted to Mogadishu Somali Turkey Training and Research Hospital's ICU.

### 2.1. ICU Admissions

The criteria for admission to an intensive care unit were used to identify hospitalizations with ICU admissions by the medical or surgical teams of the hospital.

### 2.2. Outcomes

The primary outcome measure was short-term mortality of ICU-admitted hospitalizations, defined as the ICU admission till discharge from the ICU to inpatient.

### 2.3. Study Covariates

(a) The first covariate is demographics like age and gender, (b) the second is comorbid conditions, (c) third variable is the patients' need for hospitalization for medical or surgical procedures (based on the primary diagnosis-related grouping), (d) the fourth is organ failures, (e) the fifth is hospital length of stay (f), and the sixth is the interventions made during the ICU stay, such as intubation, hemodialysis, and so on.

### 2.4. Data Analysis

Continuous variables were reported as the mean (standard deviation (SD)) or the median, while data on categorical variables were summarized as numbers and percentages (interquartile (IQR)). Continuous variables were compared using the *t*-test, Mann–Whitney test, and Kruskal–Wallis test, as appropriate. We used the Kaplan–Meier estimator test to show the survival outcome.

## 3. Results

The majority (39.7%) of the patients were adults (aged between 20 and 39 years); 734 (67.8%) were males. 595 (54.9%) were transferred to the inpatient, while 488 (45.1%) died in the intensive care unit. The median ICU length of stay was 3 days (IQR = 5 days), and nonsurvivors had a shorter stay of one day. The median no. of organ failure is one (IQR = 1). 73.4% of patients were in the ICU for less than 6 days, whereas only 4.2% of patients were in the ICU for more than 20 days.  Table 1 describes sociodemographic and clinical characteristics among patients admitted to the ICU. When it comes to diagnosis on admission, complications related to acute and chronic kidney injuries top the list of medical patients, where perforation due to organ injury top the list of surgical patient, as Somalia is a war-torn country, where gunshot injuries are prevalent.

Table 2 shows sociodemographic factors and their association with outcome using the Chi-square test. Since the *p* values for age group, comorbidity, type of organ failure, clinical intervention, type of admission, duration, and number of organ failures were less than 0.05, we may infer that all these variables significantly affect the outcome.

**Figure 1:**
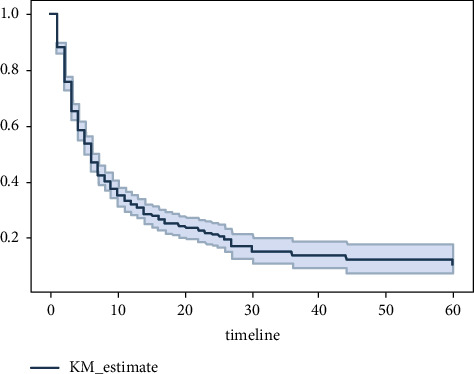
Kaplan–Meier curve for outcome variables.

**Figure 2:**
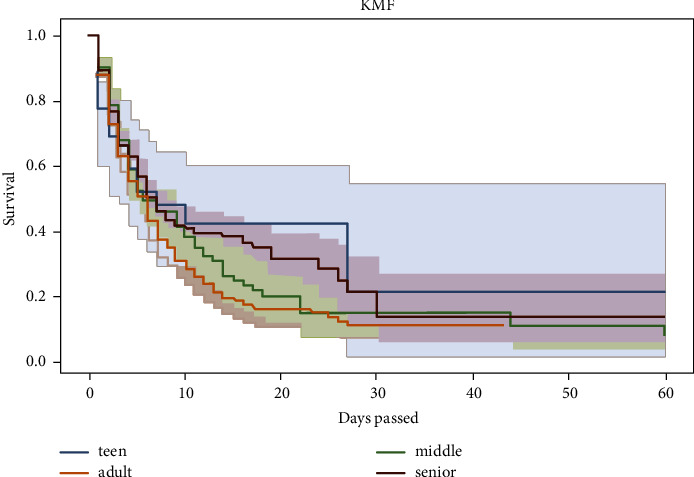
Kaplan–Meier curve for outcome variables and age group.

**Figure 3:**
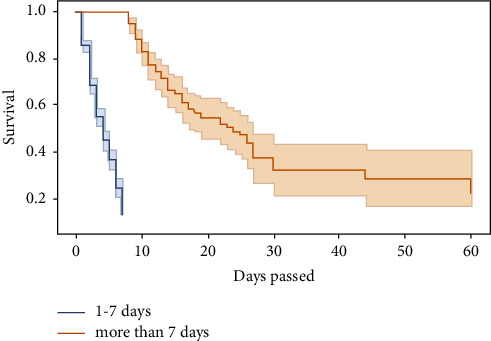
Kaplan–Meier curve for outcome variables and duration.

**Figure 4:**
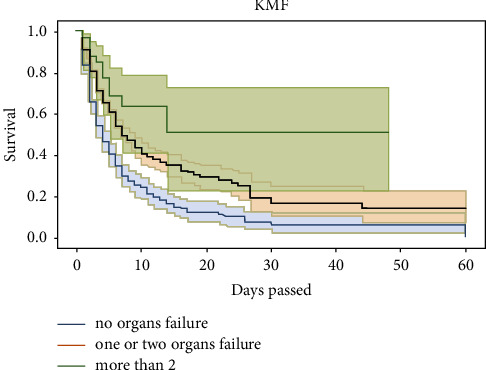
Kaplan–Meier curve for outcome variables and no. of organ failure.

The survivor function *S*(*t*) is the Kaplan–Meier estimator. From Figure 1, we can see that most patients died in the first 20 days, as indicated by the steep slope of the estimated survival function in the first 20 days (Figure 2). *S*(10) = 0.3 means that the probability of a patient surviving longer than ten days is 30%. Kaplan–Meier estimator showed that the probability of a patient surviving longer than two days in the ICU is 76%; however, the probability of surviving longer than 20 days is dropped to 23%. Survival curves do not go head-to-head, indicating that there is a difference between groups being studied (Figures 3 and 4).

The factors associated with outcome in univariate logistic regression are displayed in Table 3. It can be seen that the *p* value of hypertension, renal failure, respiratory failure, clinical intervention, and duration is below <0.005. Therefore, these variables are statistically significant. On univariate analysis, adult patients had a considerably higher likelihood of experiencing the outcome (OR = 1.707, 95% CI: 1.271–2.285). Surprisingly, compared to patients with three or more organ failures, patients without organ failure were more likely to have a outcome (OR = 4.586, 95% CI: 2.174–9.674). The outcome was significantly lower in patients with hypertension as compared to patients without hypertension (OR = 0.629 95% CI: 0.463–0.855). The patients who did not have respiratory failure were less likely to have outcome compared to the patients who had respiratory failure (OR = 1.472 95% CI: 1.111–1.950). Patients without renal failure were more likely to have outcome compared to patients with renal failure. Patients admitted through medical had lower odds of outcome than those who admitted through surgical (OR = 0.6140, 95% CI: 0.497–0.786). Patients who stay less than 7 days in ICU were less likely to have outcome in comparison with patients who stay more than 7 days (OR = 2.114, 95% CI: 1.559–2.868).

The Cox-proportional hazard model estimates are shown in Table 4. The variables respiratory, clinical intervention, intubation, and duration have highly statistically significant coefficients. The *p* value for clinical intervention is <0.005 and HR is 1.292, indicating a strong relationship between the clinical intervention and increased risk of death. HR is 1.292 for respiratory; this means that a patient's hazard ratio increases by a factor of 1.292 (versus the baseline).

## 4. Discussion

We conducted a single center based analysis of ICU-admitted patients in Mogadishu. The center has 21 tertiary level ICU beds, the only one in Mogadishu, with a population of around 2.5 million [5]. This clearly indicates the scarcity of tertiary ICU levels in Somalia.

In this retrospective study, we aimed to determine admission patterns in our ICU during the year 2021. The overall mortality rate was 45.1%, somewhat higher than the reports from the neighbouring country, Ethiopia [6]. This could be caused by patient care-seeking delays, a lack of treatment protocol, a pharmaceutical shortage, or other factors. The outcome of intensive care depends on many issues given by the surgeons and doctors who make the first judgments that lead to their patients needing intensive care, as well as the amenities offered in the unit and the competence and timing with which they are administered [7]. The length of stay was comparable to some US hospitals (3 days) [8], but lower than studies from Austria and Switzerland (7.6 in survivors and 11.7 in nonsurvivors) [9] and higher than Scandinavian countries (1.9 in nonsurvivors) [10]. Males were admitted to our ICU in greater numbers (67.8%) than females. The majority of males accessing health facilities, which is also observed in all hospital admissions as recorded by other studies in Ethiopia, could be one of the causes [11, 12]. However, this is in contrast to the general demographic trend, where women somewhat outnumber men (100 : 99.44) [13].

Identifying patients who should be targeted for interventions early in their hospital or ICU course is one of the challenges to improving the quality of end-of-life care in the ICU. There are several potential methods for identifying the most appropriate patients. The SUPPORT study, for example, is aimed at seriously ill patients with one or more of the following illnesses: acute respiratory failure, multiple organ system failure with sepsis, multiple organ system failure with malignancy, coma, chronic obstructive pulmonary disease with respiratory failure, decompensated congestive heart failure, severe cirrhosis, metastatic colon cancer, and non-small-cell lung cancer [14]. As our ICU was a level 3 ICU, most of the interventions needed to support vital organs were available such as mechanical ventilation and hemodialysis.

When it comes to the type of organ failure, renal failure topped among all, with respiratory failure being second in the rank. This is in consistent with a previous report by Sari and Bashir [15], about the patients admitted to the medical ward, which showed that 45% of the admissions were due to renal failure. However, it is in contrary to the findings of Sakr et al. from Belgium that the cardiovascular failure was the most common type of organ failure [16].

## 5. Conclusion

In this first ICU-related study from Somalia, we demonstrated the demographic characteristics of and the outcome of patients admitted to a level 3 ICU in Mogadishu. It also demonstrated the scarcity of ICU beds in Somalia's capital city of Mogadishu. Although there are facilities for tracheal intubation, mechanical ventilation, hemodialysis, and patient monitoring, the survival rate of patients in our ICU is uncomfortably low. There is a lack of data in our region on workload, outcomes, costs, and the heterogeneity of ICUs; any recommendation about future provision will be highly speculative.

## 6. Limitations

This study has limitation. Due to the retrospective nature of the study, we could not get all data relating to APACHI Score and SAPS.

## Figures and Tables

**Table 1:** Descriptive statistics for sociodemographic and clinical characteristics among patients.

Variables		Frequency	Percent
Age group (years)	16–19	38	3.5
	20–39	430	39.7
	40–59	274	25.3
	60+	340	31.4
	Total	1082	99.9
	Missing	1	0.1

Gender	Male	734	67.8
	Female	348	32.1
	Total	1082	99.9
	Missing	1	0.1

Outcome	Nonsurvived	488	45.1
	Survived	595	54.9
	Total	1083	100

Type of admission	Medical	546	50.4
	Surgical	535	49.4
	Total	1081	99.8
	Missing	2	0.2

*Diagnosis on admission*			
Medical	Acute kidney injury	167	30.5
	Chronic kidney disease	100	18.3
	Heart failure	80	14.6
	ARDS	80	14.7
	Sepsis	50	9.2
	Pulmonary edema	34	6.2
	Pneumonia	25	4.5
	Stoke	10	1.8
Surgical	Perforated viscus due to gunshot injuries	149	27.9
	Perforated appendix	105	19.6
	Sepsis	98	18.3
	Acute kidney injury	83	15.5
	Pneumonia	50	9.3
	Brain traumatic injury	49	9.2

Source of admission	Inpatient	454	41.9
	Emergency	627	57.9
	Total	1081	99.8
	Missing	2	0.2

Comorbidities	No	661	61.0
	Diabetes	188	17.4
	Hypertension	138	12.7
	Stroke	11	1.0
	Chronic kidney disease	10	0.9
	Cirrhosis	3	0.3
	Diabetes and hypertension	29	2.7
	Diabetes and stroke	1	0.1
	Diabetes and chronic kidney disease	1	0.1
	Hypertension and stroke	5	0.5
	Hypertension and chronic kidney disease	6	0.6
	Stroke and hypertension	1	0.1
	Diabetes, hypertension, and stroke	5	0.5
	Diabetes, hypertension, and chronic heart disease	3	0.3
	Total	1062	98.1
	Missing	21	1.9
Type of organ failure	No	471	43.5
	Renal failure	275	25.4
	Heart failure	38	3.5
	Respiratory failure	152	14.0
	Liver failure	5	0.5
	Renal failure and heart failure	41	3.8
	Renal failure and respiratory failure	45	4.2
	Renal failure and liver failure	4	0.4
	Heart failure and respiratory failure	15	1.4
	Renal failure, heart failure, and respiratory failure	14	1.3
	Renal failure, heart failure, and liver failure	2	0.2
	Renal failure, respiratory failure, and liver failure	5	0.5
	Heart failure, respiratory failure, and liver failure	1	0.1
	Total	1068	98.6
	Missing	15	1.4

Clinical intervention	No	369	34.1
	Dialysis	91	8.5
	Intubation	227	21.0
	Surgery	127	11.7
	Chest tube	29	2.7
	Dialysis and intubation	58	5.4
	Dialysis and surgery	6	0.6
	Intubation and surgery	142	13.1
	Intubation and chest tube	7	0.6
	Surgery and chest tube	3	0.3
	Surgery and medical	1	0.1
	Dialysis, intubation, and surgery	10	0.9
	Intubation, surgery, and chest tube	6	0.6
	Total	1076	99.4
	Missing	7	0.6

No. of organ failure	0	377	34.8
	1	467	43.1
	2	189	17.5
	3	33	3.0
	4	1	0.1
	Total	1067	98.5
	Missing	16	1.5

Total		1083	100.0

**Table 2:** Descriptive statistics for sociodemographic and clinical characteristics among patients with dead and transferred to the inpatient.

Patient's characteristics		Outcome		*P* value
		Nonsurvived	Survived	
Age group (years)	16–19	17	21	0.002^*∗∗*^
	20–39	163	267	
	40–59	133	141	
	60+	174	166	

Gender	Male	323	411	0.360^†^
	Female	164	184	

Comorbidity	Diabetes	104	84	0.006^*∗∗*^
	Hypertension	54	84	
	Stroke	6	5	
	Diabetes and hypertension	20	38	

Type of organ failure	Renal failure	148	127	0.000^*∗∗*^
	Heart failure	12	26	
	Respiratory failure	58	94	
	Renal and heart failure	45	41	
	Renal and respiratory	69	29	
	Heart and respiratory	11	327	

Clinical intervention	Dialysis	15	75	0.000^*∗*^
	Intubation	195	32	
	Surgery	15	112	
	Dialysis and intubation	95	10	
	Intubation and surgery	104	118	

Type of admission	Medical	278	268	0.000^*∗∗*†^
	Surgical	209	326	

Source of admission	Inpatient	198	257	0.421^†^
	Emergency	289	338	

Duration	<7 days	350	504	0.000^*∗∗*†^
	7 or more days	138	91	

No. of organ failures	No organ failure	121	257	0.000^*∗∗*^
	1 or 2 organ failures	335	321	
	3 or more organ failures	24	11	

^*∗*^Significant at *p* < 0.05. ^*∗∗*^significant at *p* < 0.01. ^†^Fisher's exact test is reported.

**Table 3:** Associations of factors with outcome.

Patient's characteristics		Crude OR (95% CI)^a^	*P* value
Age group (years)	13–19	1.194 (0.600–2.376)	0.615
	20–39	1.705 (1.271–2.285)	0.000^*∗∗*^
	40–59	1.145 (0.827–1.586)	0.413
	60+	Ref	

Gender	Male	1.112 (0.856–1.445)	0.426
	Female	Ref	

Comorbidity	No	1.038 (0.811–1.330)	0.766
	Yes	Ref	

Diabetes	No	1.233 (0.930–1.636)	0.146
	Yes	Ref	

Hypertension	No	0.629 (0.463–0.855)	0.003^*∗∗*^
	Yes	Ref	

Stroke	No	0.676 (0.329–1.389)	0.287
	Yes	Ref	

Renal failure	No	2.522 (1.959–3.245)	0.000^*∗∗*^
	Yes	Ref	

Heart failure	No	1.063	0.737
	Yes	Ref	

Respiratory	No	1.472 (1.111–1.950)	0.007^*∗∗*^
	Yes	Ref	

Clinical intervention	No	9.728 (6.122–15.456)	0.000^*∗∗*^
	Yes	Ref	

Dialysis	No	1.860 (1.380–2.507)	0.000^*∗∗*^
	Yes	Ref	

Intubation	No	14.086 (10.336–19.196)	0.000^*∗∗*^
	Yes	Ref	

Surgery	No	0.559 (0.430–0.726)	0.000^*∗∗*^
	Yes	Ref	

Type of admission	Medical	0.614 (0.479–0.786)	0.000^*∗∗*^
	Surgical	Ref	

Source of admission	Inpatient	1.087 (0.848–1.394)	0.511
	Emergency	Ref	

Duration	<7 days	2.114 (1.559–2.868)	0.000^*∗∗*^
	7 or more days	Ref	

No. of organ failures	No organ failure	4.586 (2.174–9.674)	0.000^*∗∗*^
	1 or 2 organ failures	2.022 (0.974–4.198)	0.059
	3 or more organ failures	Ref	

**Table 4:** Cox-proportional hazard model estimates.

Patient's characteristics		HR (95% CI)^b^	*P* value
Age group (years)	17–19	0.843 (0.597–1.189)	0.330
	20–39	1.058 (0.1–1.223)	0.445
	40–49	1.043 (0.886–1.228)	0.610
	60+	Ref	

Renal failure	No	1.120 (0.991–1.266)	0.070
	Yes	Ref	

Respiratory	No	1.283 (1.114–1.477)	0.001^*∗∗*^
	Yes	Ref	

Clinical intervention	No	1.292 (1.108–1.507)	0.001^*∗∗*^
	Yes	Ref	

Dialysis	No	1.115 (0.962–1.292)	0.149
	Yes	Ref	

Intubation	No	1.421 (1.255–1.608)	0.000^*∗∗*^
	Yes	Ref	
Surgery	No	1.025 (0.902–1.164)	0.706
	Yes	Ref	

Type of admission	Medical	0.942 (0.833–1.064)	0.336
	Surgical	Ref	

Duration	<7 days	65.258 (33.861–115.463)	0.000^*∗∗*^
	7 or more days	Ref	

No. of organ failures	No organ failure	1.440 (1.017–2.038)	0.040^*∗*^
	1 or 2 organ failures	1.181 (0.840–1.661)	0.338
	3 or more organ failures	Ref	

## Data Availability

The data used to support the findings of this study are available from the corresponding author upon request.
